# Research supporting malaria control and elimination in China over four decades: a bibliometric analysis of academic articles published in chinese from 1980 to 2019

**DOI:** 10.1186/s12936-021-03698-y

**Published:** 2021-03-20

**Authors:** Yan-Qiu Du, Guo-Ding Zhu, Jun Cao, Jia-Yan Huang

**Affiliations:** 1 Key Lab of Health Technology Assessment, School of Public Health, National Health Commission, Fudan University, 200433 Shanghai, China; 2 Jiangsu Provincial Key Laboratory of Parasite and Vector Control Technology, National Health Commission Key Laboratory of Parasitic Disease Control and Prevention, Jiangsu Institute of Parasitic Diseases, Wuxi, 214064 China; 3 Global Health Institute, Fudan University, Shanghai, 200433 China; 4 Center for Global Health, School of Public Health, Nanjing Medical University, Nanjing, 211166 China

**Keywords:** Malaria, Plasmodium, Anopheles, Academic articles, Bibliometric analysis

## Abstract

**Background:**

China has accumulated considerable experience in malaria control and elimination over the past decades. Many research papers have been published in Chinese journals. This study intends to describe the development and experience of malaria control and elimination in China by quantitatively analysing relevant research using a bibliometric analysis.

**Methods:**

A long-term, multistage bibliometric analysis was performed. Research articles published in Chinese journals from 1980 to 2019 were retrieved from the Wanfang and China National Knowledge Infrastructure (CNKI) databases. Year of publication, journal name and keywords were extracted by the Bibliographic Items Co-occurrence Matrix Builder (BICOMB). The K/A ratio (the frequency of a keyword among the total number of articles within a certain period) was considered an indicator of the popularity of a keyword in different decades. VOSviewer software was used to construct keyword co-occurrence network maps.

**Results:**

A total of 16,290 articles were included. The overall number of articles continually increased. However, the number of articles published in the last three years decreased. There were two kinds of keyword frequency trends among the different decades. The K/A ratio of the keyword ‘*Plasmodium falciparum*’ decreased (17.05 in the 1980s, 13.04% in the 1990s, 9.86 in the 2000s, 5.28 in the 2010s), but those of ‘imported case’ and ‘surveillance’ increased. Drug resistance has been a continuous concern. The keyword co-occurrence network maps showed that the themes of malaria research diversified, and the degree of multidisciplinary cooperation gradually increased.

**Conclusions:**

This bibliometric analysis revealed the trends in malaria research in China over the past 40 years. The results suggest emphasis on investigation, multidisciplinary participation and drug resistance by researchers and policymakers in malaria epidemic areas. The results also provide domestic experts with qualitative evidence of China’s experience in malaria control and elimination.

**Supplementary Information:**

The online version contains supplementary material available at 10.1186/s12936-021-03698-y.

## Background

Malaria research has been conducted since the foundation of the People’s Republic of China in 1949, and in 2017, China had zero local cases of malaria. The nationwide evolution of malaria research can be grouped into five phases, namely, the unknown transmission (1949–1959), outbreak and pandemic transmission (1960–1979), decline in transmission with sporadic cases (1980–1999), low transmission with re-emergence in central China (2000–2009), and elimination phases (2010 to present) [1, 2].

In the different phases, prevention and control strategies, such as joint malaria prevention and control strategies that lasted more than half a century and the ‘1-3-7’ malaria surveillance and response strategy, were constantly promoted as key interventions to deal with imported cases and local cases [3, 4]. The continuous evolution of such strategies ensured progress towards elimination. These strategies are documented in the form of research publications.

In the last 40 years, a large number of research articles on malaria have been published in Chinese journals. It is necessary to systematically review existing Chinese academic articles. The information and experience contained in academic research can serve as references for those areas that are still struggling with malaria, such as malaria hotspots. Although this information may not directly provide immediate solutions or strategies for other countries, it could assist researchers and policy-makers in predicting potential problems in the next phase, readjusting research directions in a shorter period of time, and reducing trial and error costs in the development of strategies and technologies. In recent years, some studies have summarized experience with this process and explore the value for other countries. However, most of these studies focus on local epidemiological data, prevention measures and effects [3, 5–7].

The aim of this research was to analyse articles published in Chinese journals using a quantitative bibliometric analysis [8]. Bibliometric analysis is widely used in various research fields (including food and medicine) [9–11]. In the malaria research field, this method has been used by researchers to analyse trends of malaria research in various countries, including China, India, and Malawi, and to document worldwide malaria vector resistance and anti-malarial drug resistance [12–16]. All such conventional bibliometric evaluations tend to analyze articles using static descriptions (which could be compared to taking a photograph) rather than dynamic comparisons (which could be compared to making stop motion animation). As a result, there is little quantitative evidence of the change process over several decades.

To fill this knowledge gap, a long-term, multistage bibliometric analysis of malaria-related academic articles published in the past 40 years was conducted to reveal changes in research and keyword themes in China; the results may reveal more detailed information than those obtained by conventional bibliometric analysis.

## Methods

**Fig. 1:**
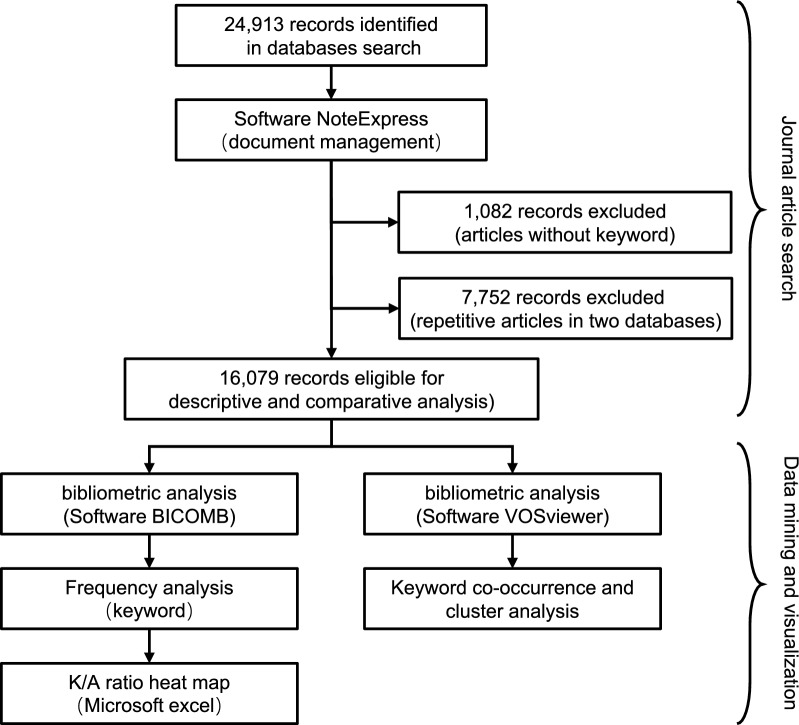
Framework of the research

The method involved in this study was mainly bibliometric analysis. The overall research framework and the software tools used are shown in Fig. 1.

### Inclusion and exclusion criteria

The search was conducted on 2 February 2020. Two major Chinese literature databases, the Wanfang and China National Knowledge Infrastructure (CNKI) databases, were searched. The inclusion criteria were as follows: Chinese articles published in journals from 1980 to 2019; and articles for which at least one of the following words was included in the title or keywords: ‘malaria’, ‘Plasmodium’, or ‘Anopheles’ [12, 13, 17]. The exclusion criteria were articles without keywords or duplicate articles in both databases. Articles with the same publication year, title, and authors were defined as duplicates in this research. NoteExpress software (Version 3.2, Aegean Technology Co. Ltd., Beijing, China) was used to manage and deduplicate the bibliographic information.

### **Data extraction**

First, all articles published within the 40-year span were analysed together, and then descriptive analyses according to criteria such as publication year, journal distribution, and highly citation rates were performed. This was followed by a comparative analysis among different decades. The 40-year span was divided into 4 time periods: the first period was from January 1, 1980 to December 31, 1989, the second period was from 1990 to 1999, the third stage was from 2000 to 2009, and the fourth stage was from 2010 to 2019. This time stratification followed the consensus of Chinese domestic malaria experts and same time periods used in other studies [1, 2]. All the bibliographic information of the included articles was exported into a format that could be analysed by bibliometric software according to time period.

### **Keyword frequency analysis** 

Bibliographic Items Co-occurrence Matrix Builder (BICOMB) software (version 2.0, School of Medical Information, China Medical University, Shenyang, China) was used to extract and analyse publication years, journal distributions, and keyword frequencies [15]. This software was developed by the Medical Information Department of China Medical University. It has good compatibility with Chinese journals and the ability to replace keywords with synonyms. For example, ‘imported case’ and ‘imported patient’ are unified as ‘imported case’.

This study defined the ‘K/A ratio’ as the frequency of a keyword among all the articles within a certain period. The absolute frequencies of keywords in different periods could not be directly compared. The K/A ratio eliminated the impact of the difference in the total number of journal articles in different periods. It was used as an indicator to compare the popularity of the same keyword in different periods. Word clouds were constructed to show the top 100 keywords in each period [18]. Microsoft Excel was used to calculate and display the change trend of the K/A ratio. A heat map was produced based on the rank of K/A ratio.

### **Keyword co-occurrence network and clustering** 

VOSviewer (version 1.6.11, Centre for Science and Technology Studies, Leiden University, Leiden, Netherlands) was used to make four keyword co-occurrence network maps for the different periods. It is a software tool that creates maps based on network data and allows the visualization and analysis of such maps [19]. This software can merge keywords with synonyms and replace Chinese with English using the ‘thesaurus terms’ file (a .txt file in a specific format for VOSviewer); such terms were translated and reviewed by two researchers. Regarding the co-occurrence analysis, if keyword A and keyword B were the keywords in one article, the relationship keywords A and B were defined as a co-occurring terms [8]. The network that was developed based on this relationship was a keyword co-occurrence network. In a network map, a node represents a keyword, and its size is related to the occurrence frequency of that keyword. The node colour represents the different cluster to which the keywords belonged. Links represent co-occurrence relationships.

For comparability among the network maps for the different periods, the parameters in VOSviewer were set as follows: the keywords with a frequency of more than 15 occurrences were shown in maps, and each cluster contained at least 5 keywords. Based on these settings, keywords were grouped into different clusters according to their co-occurrence relationship. Then, the clusters in the network maps of the four periods were compared with each other.

## Results

**Fig. 2:**
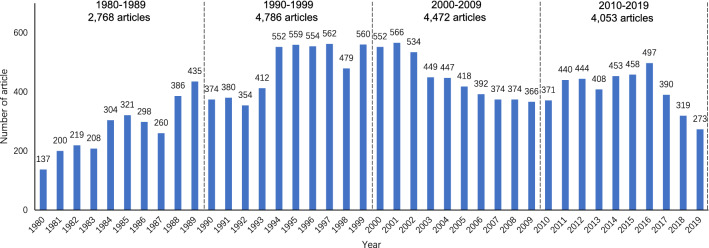
Publication trends from 1980 to 2019

In this research, 14,963 articles were retrieved from the CNKI database, and 9,950 articles were retrieved from the Wanfang database. A total of 24,913 articles were collated in NoteExpress for exclusion and deduplication. A total of 1,082 articles without keyword were excluded. Most of the excluded studies were notices, announcements and news about malaria published in academic journals. A total of 7,752 articles were excluded because they were repetitive (Fig. 2).

### Publication distribution

**Fig. 3:**
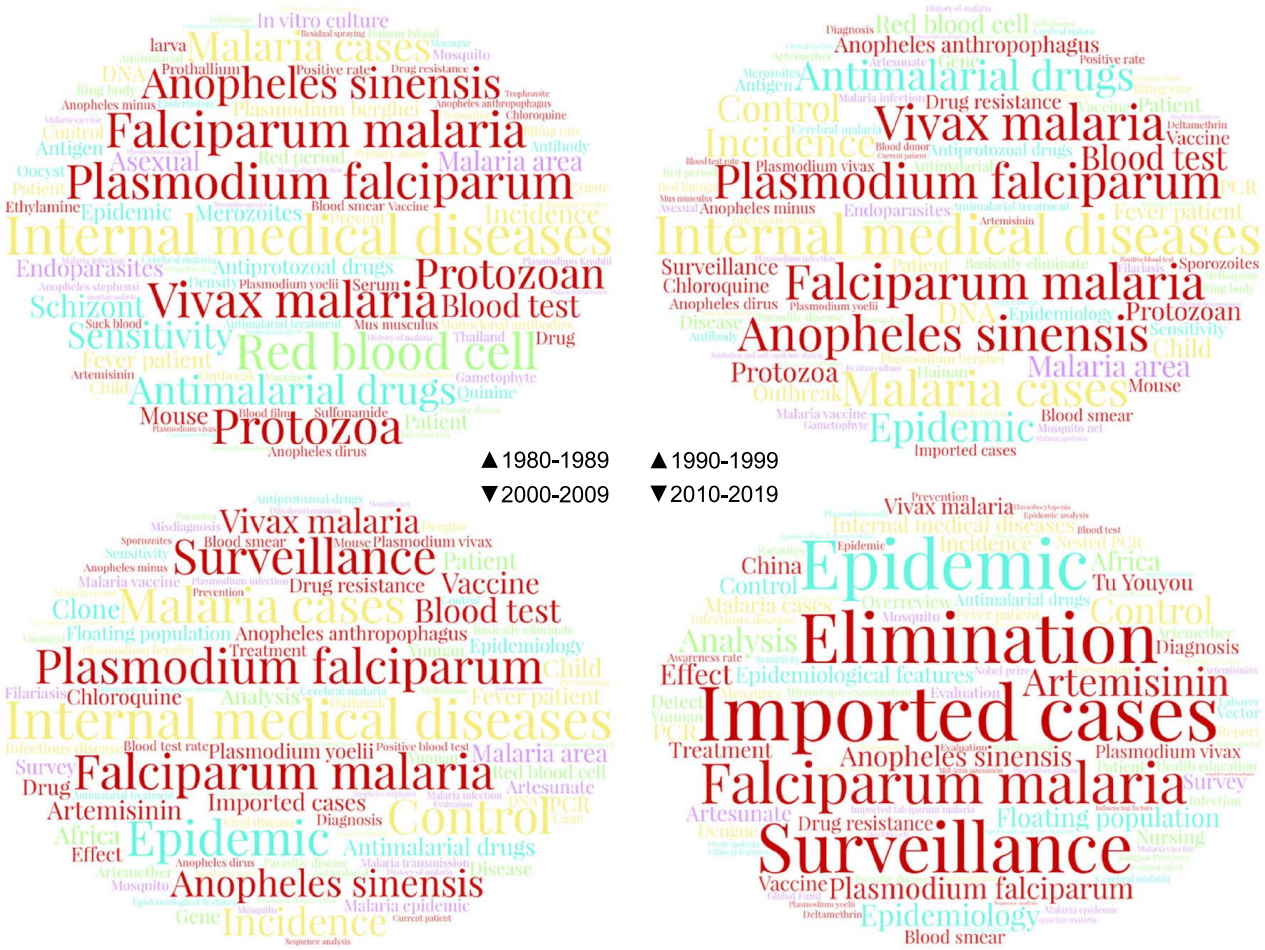
Word Clouds of the top 100 keywords in the four periods

From January 1, 1980, to December 31, 2019, a total of 16,290 articles related to malaria were published in Chinese academic journals. As shown in Fig. 3, in the 1980s and 1990s, the number of articles showed a rapid increase, from 2,768 to 4,786. There was a gradual and slight decrease from 2003 to 2010. Over the last ten years, there was a slight initial increase in the number of articles, but during the three consecutive years from 2017 to 2019, the annual decrease exceeded 10%.

The top 15 journals with the most cumulative articles published in the past 40 years are listed in Table 1. Articles related to malaria were mainly published in professional journals in the fields of parasitic diseases, tropical diseases and infectious diseases.

**Table 1:** Top 15 journals with the most publications

Rank	Journal	Records	% of total	Cumulative %
1	International Journal of Medical Parasitic Diseases	2126	13.22	13.22
2	Chinese Journal of Parasitology and Parasitic Diseases	1207	7.51	20.73
3	Journal of Parasitic Biology	1113	6.92	27.65
4	China Tropical Medicine	699	4.35	32.00
5	Chinese Journal of Schistosomiasis Control	462	2.87	34.87
6	Parasitoses and Infectious Diseases	433	2.69	37.56
7	Chinese Journal of Vector Biology and Control	275	1.71	39.27
8	Journal of Medical Pest Control	274	1.70	40.98
9	Chinese Journal of Zoonoses	240	1.49	42.47
10	Journal of Tropical Medicine	197	1.23	43.70
11	Henan Journal of Preventive Medicine	192	1.19	44.89
12	Hainan Medical Journal	166	1.03	45.92
13	Acta Parasitological et Medica Entomological Sinica	138	0.86	46.78
14	Chinese Journal of Public Health	131	0.81	47.60
15	Modern Preventive Medicine	126	0.78	48.38

International Journal of Medical Parasitic Diseases was suspended in 2015

### Highly cited articles and authors

The highly cited articles from each period that were extracted from the CNKI database are shown in an Additional file (see Additional file 1). The deadline for citation analysis was February 2, 2020. The citation frequencies of highly cited articles varied greatly in each period, and the themes represented by articles also varied greatly. In the 1980 s, the most-cited articles mainly focused on Anopheles. In the 1990s, the number of themes increased, but anti-malarial drugs and vectors were still the main themes. In the 2000s and 2010s, citations of epidemic analysis publications increased, and in the last ten years, retrospective and summary research articles received increasing attention. Most of the authors of the highly cited articles were from national or provincial institutes of parasitic diseases and Centers for Disease Control and Prevention. Regarding the frequency of author affiliations, the top 5 institutions in the different periods are shown in Table 2. National and provincial institutes of parasitic diseases occupied most of the top 5 positions in the 1990s, 2000s and 2010s, followed by universities.

**Table 2:** Top 5 institutions with the most author frequency

Years	No.	Institute	Frequency
1980s	1	Henan Provincial Health and Anti-epidemic Station	36
	2	Yunnan Institute of Parasitic Disease	30
	3	Liaoning Provincial Health and Anti-epidemic Station	15
	4	Sun Yat-sen University	12
	5	Hainan Provincial Health and Anti-epidemic Station	11
1990s	1	Guangdong Institute of Parasitic Disease	47
	2	Shandong Institute of Parasitic Disease	29
	3	Sun Yat-sen University	29
	4	Hainan Institute of Parasitic Disease	28
	5	Yunnan Institute of Parasitic Disease	23
2000s	1	Yunnan Institute of Parasitic Disease	80
	2	National Institute of Parasitic Disease	77
	3	Sun Yat-sen University	48
	4	China Medical University	47
	5	Third Military Medical University	44
2010s	1	National Institute of Parasitic Disease	94
	2	China Medical University	62
	3	Second Military Medical University	42
	4	Guangxi Center for Disease Control and Prevention	30
	5	Chinese Academy of Medical Sciences	28

### Keyword frequency analysis 

The word clouds for the different periods (Fig. 3) revealed the following features: (1) falciparum malaria and vivax malaria were the main types of malaria in China; (2) *Anopheles sinensis* was the main malaria vector; and (3) imported cases, surveillance and elimination had become great concerns in the fourth stage.

In the analysis of single keywords, the keyword ‘*Plasmodium falciparum*’ was ranked fourth in the first three periods, but its K/A ratio declined over time, at 17.05% in the 1980s, 13.04 in the 1990s, and 9.86% in the 2000s. The frequency rank of the keyword ‘falciparum malaria’ increased from seventh in the 1980s to fourth in the 2010s, but its K/A ratio declined from 12.97% in the 1980s to 7.82 % in the 2010s. These results suggested that there were some common patterns in the changes in K/A ratios.

**Fig. 4a:**
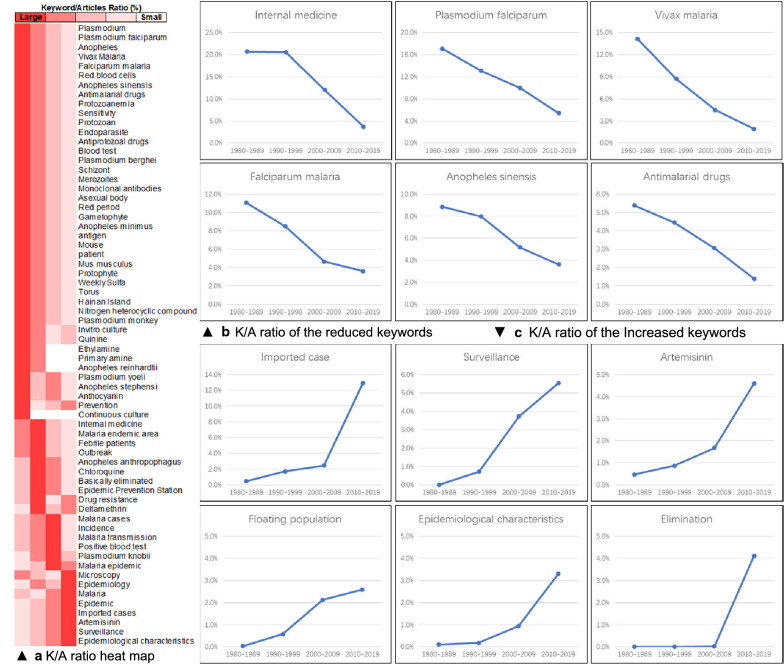
K/A ratio heat map,** b** K/A ratio of the decreased keywords,** c** K/A ratio of the increased keywords

The heat map (Fig. 4a) showed two obvious patterns in the overall change in the K/A ratio. It clearly showed that the K/A ratios of some keywords continuously decreased, while those of other continuously increased (Fig. 4b, c). Keywords with continuous decreases in their K/A ratios were ‘internal medicine’, ‘*Plasmodium falciparum*’, ‘vivax malaria’, ‘falciparum malaria’, ‘*Anopheles sinensis*’, and ‘antimalaria drugs’. Keywords with continuous increases included ‘imported case’, ‘surveillance’, ‘artemisinin’, ‘floating population’, ‘epidemiological characteristic’, and ‘elimination’. In essence, these two patterns were the manifestations of changes in research themes.

### **Keywords co-occurrence network** 

**Fig. 5:**
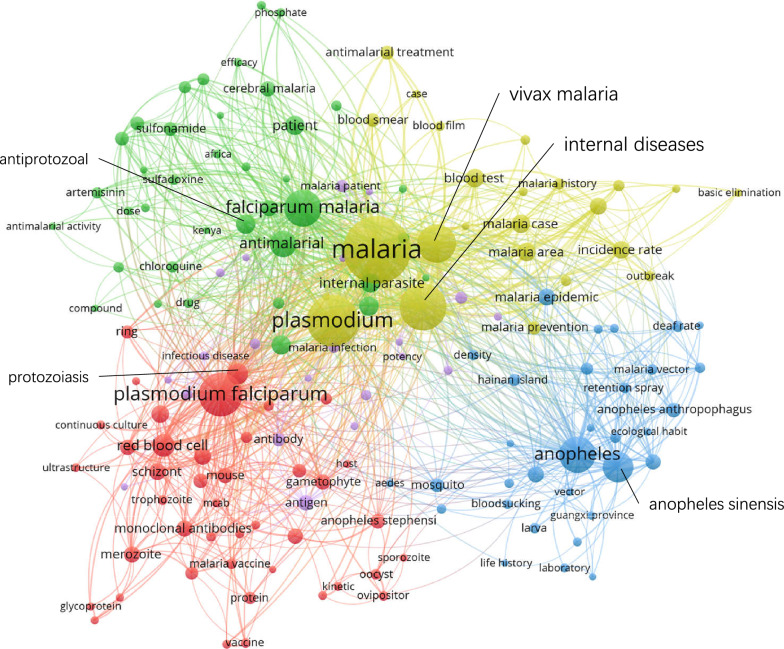
Keyword co-occurrence network map (1980–1989)

**Fig. 6:**
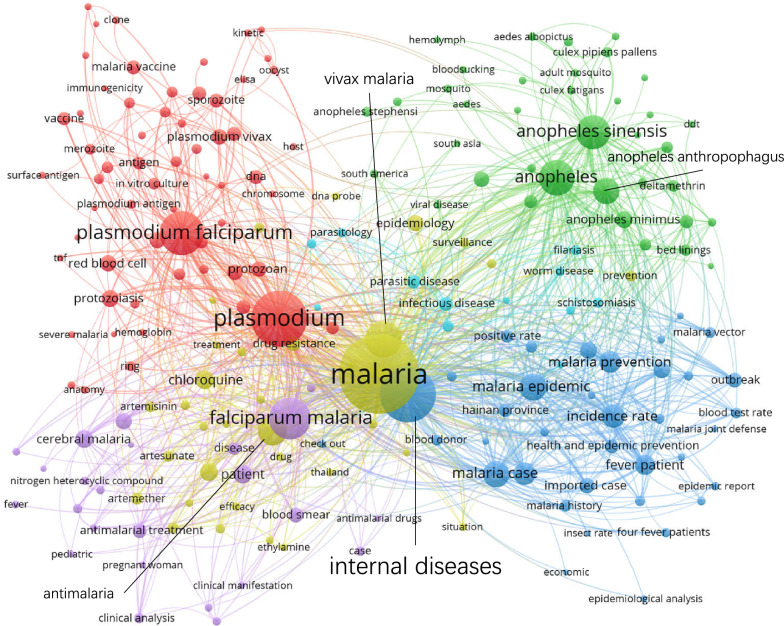
Keyword co-occurrence network map (1990–1999)

**Fig. 7:**
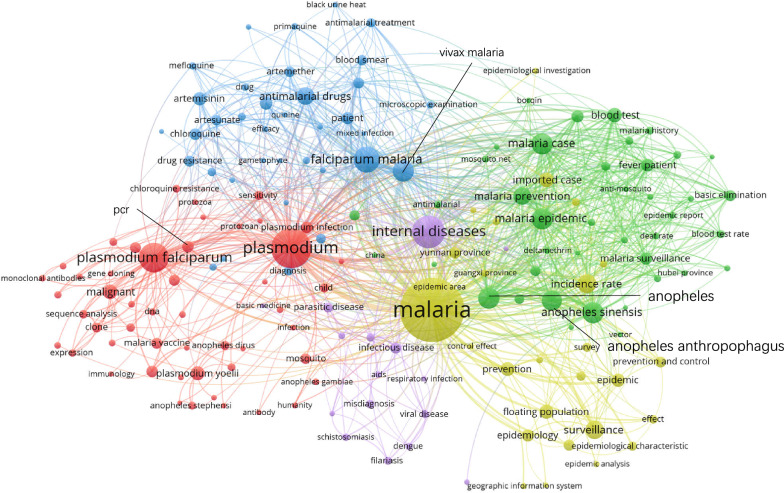
Keyword co-occurrence network map (2000–2009)

**Fig. 8:**
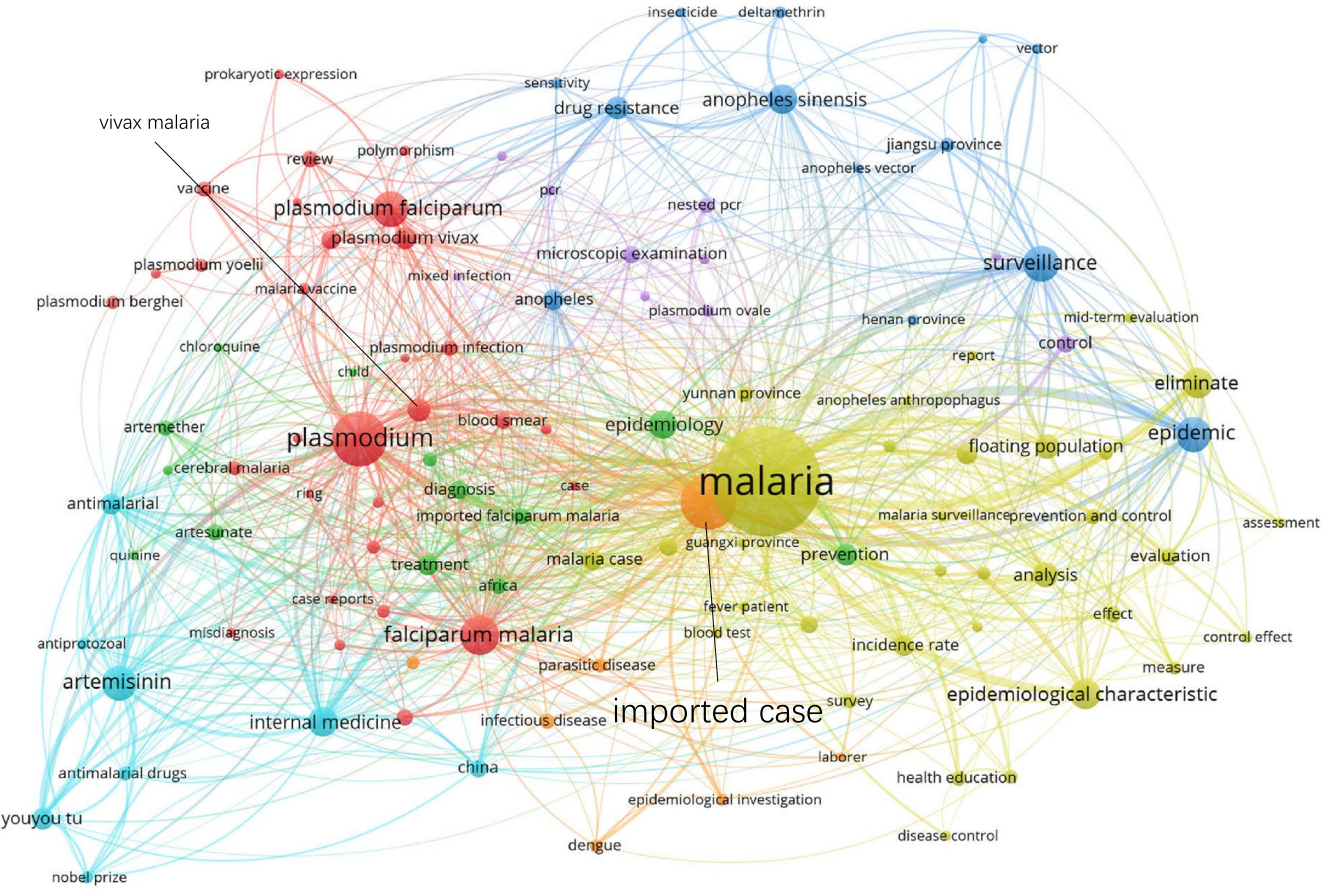
Keyword co-occurrence network map (2010–2019)

Figures 5, 6, 7 and 8 show the maps of the keyword co-occurrence networks in the four periods. According to the strengths of the co-occurrence relationships, 157 keywords were divided into 5 clusters in the 1980s, 205 keywords into 6 clusters in the 1990s, 170 keywords into 5 clusters in the 2000s, and 114 keywords into 7 clusters in the 2010s (for more information, see Additional file 2). These clusters were considered research themes, and each theme was divided into subthemes according to the subjects that were represented by specific keywords in the cluster.

Regarding the whole network maps for the four periods, in the 1980s (Fig. 5), the blue cluster was centred on ‘*Anopheles*’ and included the keywords ‘*Anopheles sinensis*’, ‘*Anopheles anthropophagus*’ and ‘*Anopheles minimus*’. The same situation occurred in the 1990s (Fig. 6, green cluster). These two clusters had obvious differentiation from other clusters within their networks. This result indicated that studies on *Anopheles* had a high degree of independence. However, in 2000s, the degree of independence decreased. By the 2010s (Fig. 8), independence had disappeared. The boundaries of the clusters were difficult to identify. In the analysis of the network structure among the clusters, the boundaries between two clusters within a period became increasingly obscured, especially in the 2010s (Fig. 8). Figure 8 also shows that many of the main nodes in a particular cluster are also intermediaries in other clusters. This result suggested that the relationship between research themes was no longer weak due to past sub-theme co-occurrence, and a strong connection emerged from the deep integration of subjects and research methods.

Regarding the clusters in different network maps, in the blue cluster in the 1980s (Fig. 5), the peripheral keywords around the central keyword ‘*Anopheles sinensis*’ included ‘retention spray’, ‘ecological habit’, and ‘life history’. However, in the blue cluster in the 2010s (Fig. 8), the peripheral keywords around the central keyword ‘*Anopheles sinensis*’ included ‘surveillance’ and ‘drug resistance’. It was found that keywords that represented the research object, such as ‘falciparum malaria’, ‘*Plasmodium falciparum*’, and ‘*Anopheles sinensis*’, were always the central keywords in different stages. However, peripheral keywords, which represented the research fields, around central keywords changed. For ‘*Anopheles sinensis*’, in the 1980s, the research direction was entomology. In the 2010s, the research direction was insect vector control. This result indicated that the research direction around the central keywords changed with the process of malaria elimination.

Regarding analysis at the node level, in all four keyword co-occurrence network maps, some nodes in one cluster co-occurred with only the nodes inside the same cluster, and other nodes co-occurred with multiple nodes outside the cluster. Under this common feature, there were differences in details, such as the link densities between nodes. In Fig. 7, the green cluster network was clearly more complex than the red cluster network even though the number of nodes was not much different between the red clusters and the green clusters. This result indicated that co-occurrence among the nodes in the green cluster was more divergent, while co-occurrence among the nodes in the red cluster was more directional. This indicated that the subthemes represented by the nodes in the red cluster had a high degree of independence.

## Discussion

In summary, the changes in the high-frequency keywords and networks reflected the research needs and hot topics in the different periods. In the 1980s, and 1990s, research topics focused on drug development and vector control. In the 21st century, with the reduction in endemic areas, formulating local strategies to deal with sporadic cases became the focus of work. Therefore, malaria surveillance reports during this period are important references. In recent years, the research theme obviously shifted towards surveillance, tracking and control of imported cases. Studies on these topics provided support for prevention and control strategies. This research revealed four long-term trends in Chinese publications. Academic research conducted at domestic institutes of parasitic diseases never stopped from the control to elimination phase. The change in the research theme was related to the need for malaria control (elimination) in the different periods. Multidisciplinary participation became popular, especially after local transmission was interrupted. Drug resistance was always a focus.

However, these trends were difficult to compare with those in other countries. There were only two bibliometric analysis publications on malaria research at the same scale in journals: Upasana’s research in India from 1909 to 2019 [12] and Chikondi’s research in Malawi from 1984 to 2016 [13]. Both studies described and analysed the research period as a whole, and there were no time-stratified comparisons. However, the author affiliation results in Chikondi’s research could be compared with the results in this research. In Malawi, the Malawi-Liverpool Wellcome Trust Programme, a politically and financially independent global charitable entity, was the most frequent author affiliation (30%), followed by the University of Malawi (20 %). In China, national and provincial institutes of parasitic diseases, as public institutions fully funded by the government, were the most frequent author affiliations. This difference could be explained by the fact that some government agencies in China were involved in malaria research and participated in the management and daily operation of public health programs. This organizational situation shortened the time required to transform research data into national or local strategies. Consistencies between research themes and national strategic plans, such as the National Malaria Control Plan (5 government documents from 1983 to 2015) and the China Malaria Elimination Action Plan (2010–2020) were clearly observed.

### Publication declines in chinese journals and international journals

In this study, a sharp decline in the number of Chinese articles published in recent years (more than 10 % per year) was observed. There was also a decline in the number of articles with Chinese participation published in international journals in 2019 [20]. With the elimination of malaria in China, the decline in malaria research was an inevitable trend. However, for the global elimination of malaria, how to fully benefit from the work of domestic researchers with extensive experience should be considered by policymakers. Chinese researchers need to actively seek out malaria-related research on a global scale, develop transnational cooperative programs to eliminate malaria, and carry out health assistance projects in developing countries.

### Continuous epidemiological investigations and surveillance

The highly cited articles and top 100 keywords among the four stages indicate that despite the control or elimination phase, epidemiological investigations and surveillance have been given considerable attention in China. Epidemiological investigations and surveillance help epidemiologists obtain basic information about malaria cases and *Plasmodium* characteristics, such as the breeding requirements of the vector, the source of imported cases, and the possibility of retransmission [4]. This information helps in the development of targeted interventions. In the 1980s, the main subjects of the investigations were vectors and transmission patterns [21–23]. In 2001, nationwide epidemiological investigation reports of malaria cases began to be published [24]. In the 2010s, epidemiological investigations of imported cases were implemented in many provinces and border regions [25–28]. Although the subjects of the investigations changed, the emphasis on epidemiological investigation has never waned. This reflects the fact that the development, adjustment, and termination of China’s antimalarial strategies were based on local epidemics and other evidence.

### The need for multidisciplinary participation

The diversification of keyword co-occurrence networks indicated a general improvement in multidisciplinary participation, which is needed to optimize large-scale social mobilization. Malaria elimination is a long-term project that requires continuous input, even when the epidemic trend has been reduced from sporadic to no local transmission. Large-scale social mobilization is the most valuable and sustainable input.

In China, social mobilization involves not only collaborative prevention and control strategies among different regions [29, 30] but also cooperation between different administrative departments, such as the Ministry of Health, Ministry of Commerce, and Inspection and Quarantine Bureau. Highly precise case tracking and patient management are needed as regions get closer to elimination. The greater the need for preventive measures for a wider range of healthy people is, the higher the cost is. Ensuring that large-scale social mobilization is efficient and precise is challenging. To solve this problem, multidisciplinary participation by people in the fields of social management, education, journalism and communications, healthcare program evaluation, and international relations is required. Multidisciplinary participation would help in the design and implementation of more efficient, low-cost and targeted interventions on the bases of multiple nodes in the transmission path. Therefore, large-scale social mobilization and multidisciplinary participation in the development of malaria strategies should be encouraged in other regions as much as possible.

## Limitations

This study has two limitations. First, the Chinese journal databases do not allow users to download reference information. Therefore, a cocitation analysis was not performed. Second, this study focused on comparisons among different periods. The full details within a single period are not presented. Many subtopic analyses can be performed. Therefore, the authors plan to share the original data (the bibliographic information of 16,290 articles) analysed in this study with other researchers to jointly evaluate these data and explore their potential value.

## Conclusions

This study revealed trends in research themes and topics in the malaria research field over the past 40 years. Revelation of these trends could help researchers in other malaria epidemic areas fully understand the research processes associated with malaria elimination strategies and technology. The results also provide domestic experts with qualitative evidence of China’s experience with malaria control and elimination. Importantly, this research promotes awareness of the importance of investigation, surveillance, and multidisciplinary participation among researchers and policy makers.

## Supplementary Information

**Additional file 1: ** Top 10 articles with high citation frequencies for the four periods.

**Additional file 2: ** Detailed information about the maps of keyword co-occurrence network output by VOSviewer.

## Data Availability

The datasets analyzed during the current study are available from the corresponding author upon reasonable request.

## Abbreviations

BICOMB: Bibliographic items co-occurrence matrix builder
CNKI: China national knowledge infrastructure
K/A ratio: Keyword/articles ratio
